# MicroRNAs: new players in IBD

**DOI:** 10.1136/gutjnl-2014-307891

**Published:** 2014-12-04

**Authors:** R Kalla, N T Ventham, N A Kennedy, J F Quintana, E R Nimmo, A H Buck, J Satsangi

**Affiliations:** 1 Gastrointestinal Unit, Centre for Molecular Medicine, Institute of Genetics and Molecular Medicine, Western General Hospital, Edinburgh, UK; 2 Centre for Immunity, Infection and Evolution, Ashworth laboratories, University of Edinburgh, Edinburgh, UK

**Keywords:** ULCERATIVE COLITIS, INTESTINAL TRACT, CROHN'S DISEASE, CELLULAR IMMUNOLOGY, T LYMPHOCYTES

## Abstract

MicroRNAs (miRNAs) are small non-coding RNAs, 18–23 nucleotides long, which act as post-transcriptional regulators of gene expression. miRNAs are strongly implicated in the pathogenesis of many common diseases, including IBDs. This review aims to outline the history, biogenesis and regulation of miRNAs. The role of miRNAs in the development and regulation of the innate and adaptive immune system is discussed, with a particular focus on mechanisms pertinent to IBD and the potential translational applications.

## Introduction

The IBDs, Crohn's disease (CD) and UC affect an estimated 2.5–3 million people in Europe, with the associated annual healthcare costs amounting to approximately €4.6–5.6 billion.^1^ The increasing incidence of early onset disease in the developed world and of disease in all ages in the developing world has catalysed studies attempting to characterise pathogenic mechanisms. In the last two decades, international collaborations have been successful in identifying susceptibility genes for IBD through genome-wide association studies (GWAS) and subsequently meta-analysis of GWAS and Immunochip data (http://www.ibdgenetics.org).^2^ These studies have been important in highlighting mechanistic pathways, notably autophagy and innate immunity in CD and epithelial barrier dysfunction in UC and have provided clues into new therapeutic strategies.

**Figure 1:**
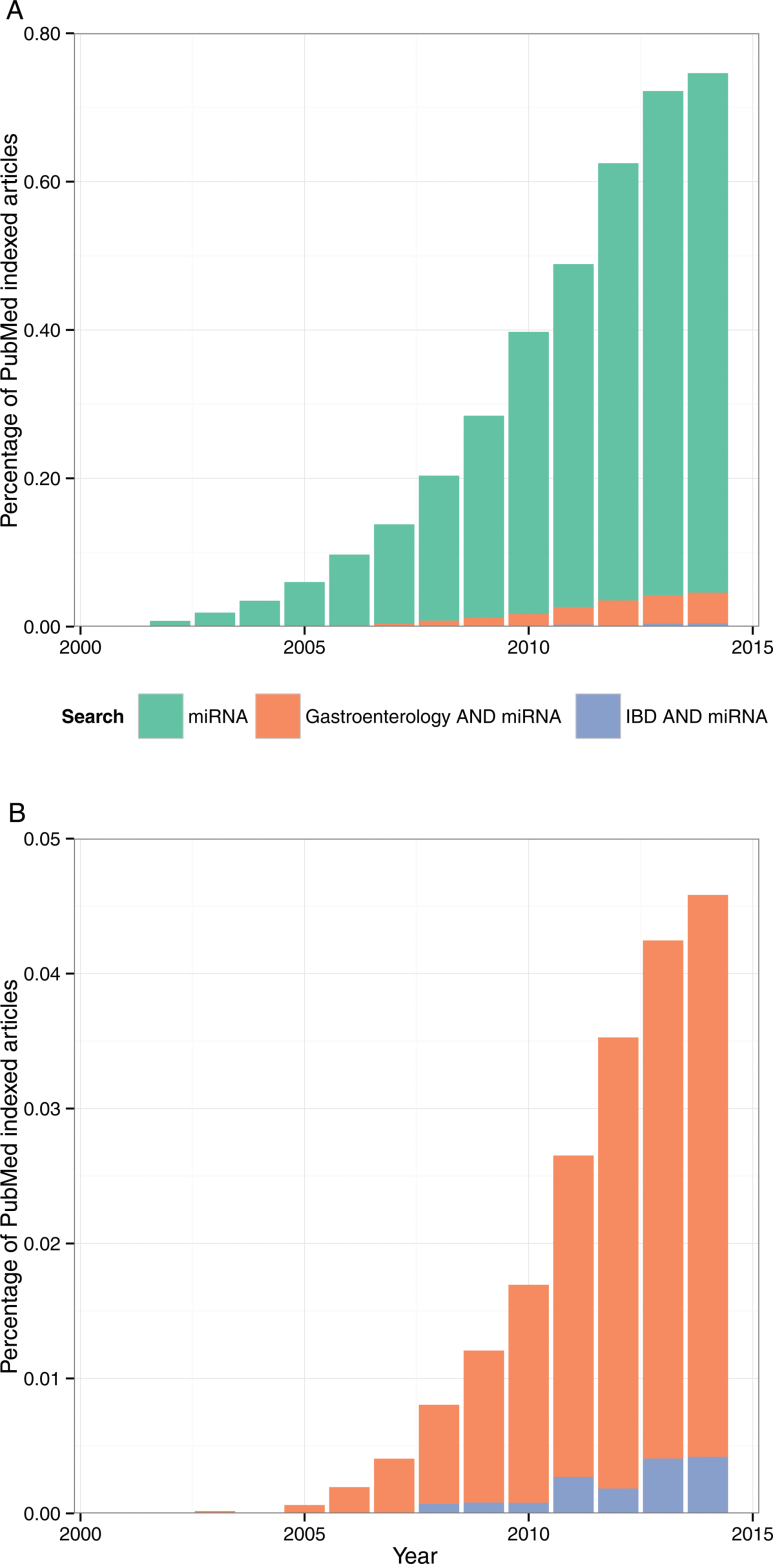
Pubmed microRNA (miRNA) citations in Gastroenterology and Inflammatory Bowel Diseases (IBD). Search terms used were as follows: *Gastroenterology*: (miRNA OR MicroRNA) AND (Gastroenterology OR IBD OR Inflammatory Bowel Disease OR Crohn’s Disease OR Ulcerative Colitis OR Colon OR Stomach OR Intestine OR Oesophagus OR Oesophagus OR Rectum) NOT mirna[author]; *IBD*: (miRNA OR MicroRNA) AND (IBD OR Inflammatory Bowel Disease OR Crohn’s Disease OR Ulcerative Colitis) NOT mirna[author]; *miRNA*: (miRNA OR MicroRNA) NOT mirna[author]; Each search was run for print publication dates for each year from 2001 to 2014. Citations were normalised to the total number of Pubmed indexed articles during the same time period (nb, the term microRNA was introduced in 2001).

There is now increasing interest in exploring epigenetic mechanisms in common diseases, with notable progress in studies of DNA methylation, histone modifications, long intergenic non-coding RNAs and in characterising the contribution of microRNAs (miRNAs). miRNAs are short strands of non-coding RNA (∼22 nt long) encoded in genomic DNA which post-transcriptionally regulate expression. The field of miRNA research is expanding rapidly with the number of miRNA-related citations increasing almost exponentially (figure 1) and miRNAs have been implicated in neurological diseases, cardiovascular diseases, autoimmune diseases and cancer.^3^ With such a wealth of data now available, reviews have been published on individual miRNAs in health and disease. miR-21 is perhaps the most compelling miRNA involved in IBD, with associations between miR-21 and IBD being replicated in several studies, functional relevance in mouse models, as well as being highly expressed in other diseases including cancer. Key miRNAs, such as miR-21, are the focus of anti-miR therapeutic development.^4–8^

Well-designed high-impact publications have established functional interactions between miRNAs and key mechanisms implicated by GWAS in IBD, notably T helper cell (Th)17 mediated inflammation and autophagy.^9^ ^10^ The review aims to outline the history, biogenesis and regulation of miRNAs. The important role of miRNAs in the development and regulation of an innate and adaptive immune system is discussed, with a particular focus on IBD pathogenesis and other immune-mediated diseases. The review will also provide an insight into the translational applications of miRNAs as biomarkers and the potential therapeutic miRNA application.

### MicroRNAs: a historical perspective

miRNAs were first identified in 1993 in the nematode model organism (*Caenorhabditis elegans*) using a genetic screen to identify defects in postembryonic development.^11–13^ It became evident that lin-4, which emerged as the first described miRNA, was able to downregulate a nuclear protein called lin-14, thereby initiating the second stage in larval development.^13^ ^14^ By the turn of the century a second miRNA, let-7, was identified in *C. elegans* that appeared to be highly conserved among species including humans.^15^ ^16^ At the time of writing 35 828 mature miRNAs occurring across all species have been registered in miRbase (http://mirbase.org, Release 21, accessed June 2014).^17^

### Biogenesis of microRNAs

**Figure 2:**
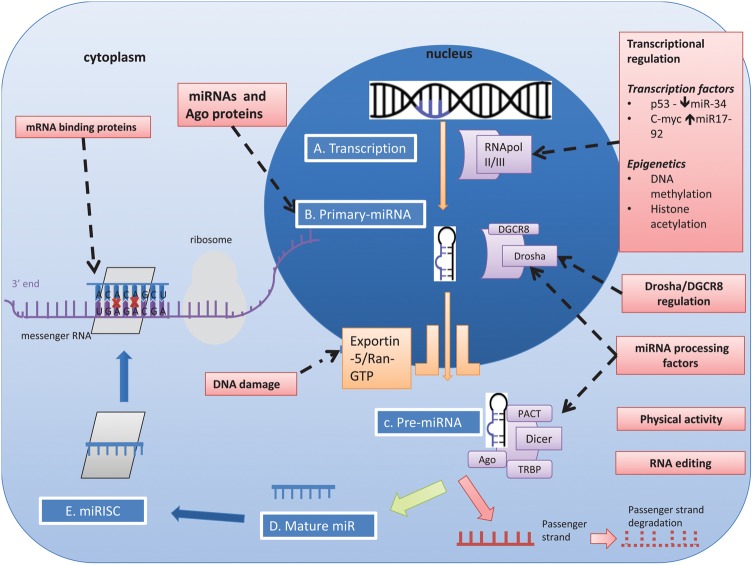
**miRNA biogenesis and regulation.** (A) Processing begins in the nucleus where primary miRNA transcripts (pri-miR) are transcribed by RNA polymerase II or RNA polymerase III.^21^ ^22^ (B) Nuclear cleavage of pri-miRNA is performed by a protein complex consisting of the RNAse-III-type enzyme Drosha and DGCR8 (DiGeorge critical region 8), which generates a 60–70 nucleotide sequence called pre-miRNA. Drosha cleavage generates a 2 nucleotide 3' overhang which appears to be a key biogenesis step.^23^ DCGR8 acts as an anchor on the stem loops of the target miRNA,^24^ allowing Drosha to correctly position on the pri-miRNA.^25^ Mirtrons are similar in structure but do not undergo Drosha/DGCR8 processing. (C) pre-miRNA is transported from the nucleus to the cytoplasm by the Exportin-5 (Exp5) — RanGTP complex. Correct binding of the double stranded stem and 3' regions to the RanGTP structure stabilises the miRNA, preventing degradation and facilitating the correct transport of pre-miRNA.^26^^–^28 (D) Final cleavage of the hairpin loop is performed by Dicer (RNAse III like enzyme) with co-factors: Tar RNA binding protein (TRBP); and protein activator of double-stranded RNA-dependent protein kinase (PACT). (E) The remaining 22 nucleotide RNA duplex is incorporated with Ago proteins, forming a pre-RNA induced silencing complex (pre-RISC). The duplex is separated within Ago proteins into a single stranded mature miRNA and its passenger strand. The mature miRNA strand is retained to form RISC which is eventually destined for mRNA repression/cleavage while its passenger strand undergoes degradation.^29^ ^30^ miRNA recognises its target via 6-8 nucleotide sequence at the 5' end of the miRNA however the binding site can vary. **Examples of regulatory elements in miRNA biogenesis**. **Transcriptional regulation** Transcription factors can influence miRNA expression by binding directly to promoter elements. Examples include c-Myc binding and upregulating miR-17–92 cluster and p53interaction with miR-34.^31^^–^34 **miRNAs and argonaute (Ago) proteins as regulators** mature miRNAs can act as regulators of miRNA processing either as an auto-regulatory loop or for other miRNAs (e.g. the biogenesis of let-7).^35^ **RNA editing** Once transcribed, miRNAs can undergo editing, which can influence miRNA target specificity.^36^^–^39 RNA editing occurs in ∼6% of human miRNAs with some studies reporting higher levels of RNA editing (50%).^37^ ^40^ RNA editing is miRNA gene- and tissue-specific (e.g A to I edited members of the miR-376 family specifically within the human cortex).^38^ ^40^ **Drosha/DGCR8** The Drosha-DGCR8 complex can undergo post-transcription self-regulation, which allows circulatory negative feedback once sufficient microprocessor activity is available.^41^^–^43 Cross-regulation between Drosha and DGCR8 may assist in homeostatic control of miRNA biogenesis.^42^ **miRNA processing factors** Specific proteins can either directly or indirectly up-regulate or downregulate the maturation of select miRNAs. A nucleo-cytoplasmic protein with dual functionality is heterogeneous nuclear ribonucleoprotein A1 (hnRNP-A1) which facilitates nuclear pri-miR-18a processing.^44^^–^47 **Physical activity** - Physiological changes such as exercise can induce modifications in the miRNA biogenesis machinery. Following 3 hours of endurance exercise in an untrained male, there is upregulation of Drosha, Dicer and Exp5 mRNA levels.^48^ **DNA damage** - DNA damage can promote post transcriptional processing of primary and precursor miRNAs which play a role in the initiation, activation and maintenance of the DNA damage response.^49^ DNA damage accelerates nuclear export of pre-miRNAs via Exp5- nucleopore-Nup153 interaction.^50^ **mRNA binding proteins** - mRNA binding proteins bind to the 3-UTR elements of the target mRNA and can either enhance or reverse translational repression by influencing mRNA-miRNA complex interaction.^51^ ^52^

miRNA genes are located throughout the genome, either within intronic sequences of protein-coding genes, within intronic or exonic regions of non-coding RNAs, or set between independent transcription units (intergenic).^18^ Some miRNAs have their own promoters and are transcribed independently, some share promoters with host genes,^19^ while others are co-transcribed as a single primary miRNA transcript.^20^ The biogenesis of miRNAs from transcription in the nucleus to generation of the mature miRNA in the cytoplasm is described in figure 2.

In plants, fully complementary binding occurs when the ‘seed’ region (located near the 5′end) of the miRNA binds to the 3′ untranslated region (UTR) of the target mRNA and this is sufficient for mRNA degradation to occur. In contrast, in humans, miRNAs bind to mRNA targets with incomplete complementarity, which results in mRNA destabilisation and translational inhibition.^53^ Other regions of the mRNA can also contain functional miRNA binding sites, including the 5′UTR and the amino acid coding sequence. Furthermore, beyond seed site pairing, the centre and the 3′end of the miRNA sequence can contribute to target recognition.^54–56^ Under certain conditions such as cell cycle arrest, miRNAs can alter their regulatory role from translational inhibition to upregulation of translation of target mRNAs.^57^ Studies have also shown that miRNAs influence gene expression at the post-transcriptional level, and may interfere with the process of transcription.^58^

Single nucleotide polymorphisms (SNPs) in pre-miRNA sequences are rare, occurring in only 10% of all human pre-miRNAs, and less than 1% of miRNAs have SNPs in their functional seed region.^53^ Therefore functional mutations in miRNAs are unlikely to be tolerated and negative selection may occur at these loci.

### miRNAs affect gene expression

It is estimated that miRNAs regulate more than 60% of protein coding mRNAs.^59^ Each miRNA can target hundreds of mRNAs resulting in mRNA destabilisation and/or inhibition of translation. Generally, the overall effect on target protein levels is subtle and can be thought of as ‘fine-tuning’ of cellular mRNA expression within a cell.^60^ ^61^ The combinatorial targeting of genes by miRNAs in this fashion makes them interesting therapeutic candidates that in theory may reduce resistance in diseases such as cancer.^62^

miRNAs regulate important cellular functions such as differentiation, proliferation, signal transduction and apoptosis and exhibit highly specific regulated patterns of gene expression.^63^ A number of applications have been developed to predict mRNA/miRNA interactions and aid in understanding specific miRNA targets.^64^

### miRNA regulation

At various stages in miRNA biogenesis, several factors can influence the development of the mature miRNA. Figure 2 depicts the various steps of biogenesis that are subject to regulation. These include regulation of transcription, cleavage of the stem loop structures by Drosha and Dicer enzymes, editing as well as regulation of miRNA turnover. The regulatory mechanisms occurring at each stage have been reviewed elsewhere.^18^ ^65^ Each of these mechanisms acts as part of a signalling network that modulates gene expression in response to cellular or environmental changes.

### miRNA gene regulatory networks

**Figure 3:**
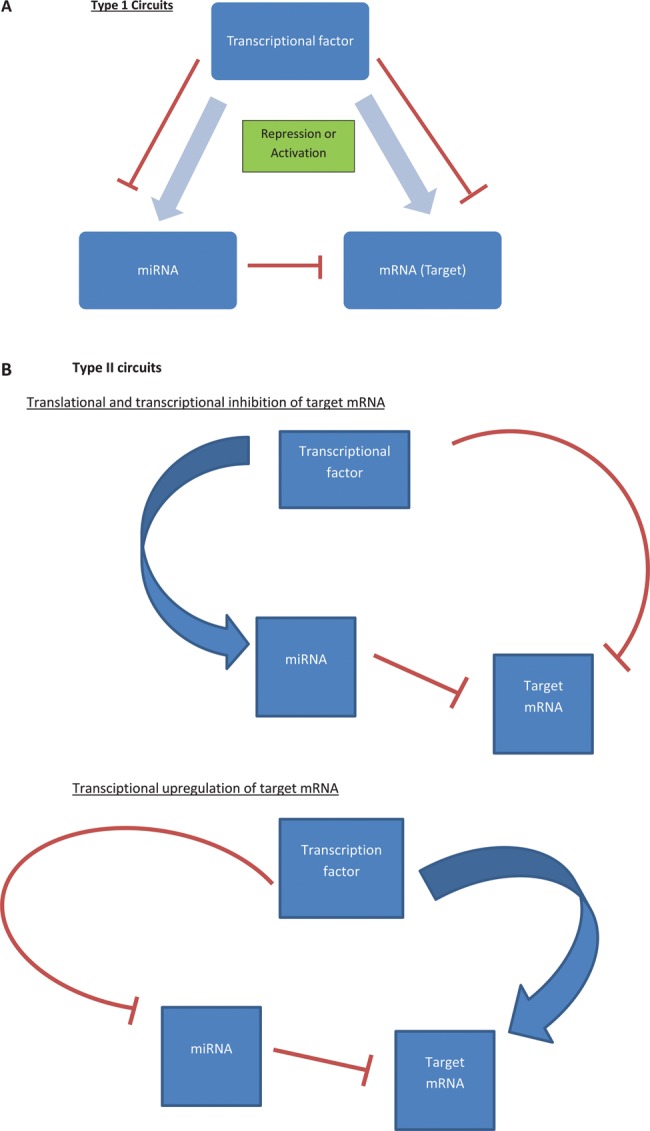
Examples of miRNA circuits. Tsang and Milo describe two distinct circuits, Type I and Type II that incorporate miRNAs in their regulatory machinery.^66^ ^67^ (A) In Type I circuits, upstream transcription factors will positively coregulate miRNA and their target mRNA.^66^ One such example is the repression of E2F1 by miR-17-5p, both of which are activated by the transcription factor c-Myc.^68^ It has been suggested that the function of such circuits is to define and maintain target-protein homoeostasis, especially in cells that are ultrasensitive to target mRNA abundance.^66^ (B) Type II circuits allow transcriptional activation or repression (positive or negative feedback loop) of a target gene by an upstream factor with associated synergistic miRNA expression.^66^ If an mRNA is to be repressed, transcription factors will downregulate the mRNA directly and also upregulate the relevant miRNA. If however a mRNA is to be upregulated, this would occur directly by the transcription factor with synergistic miRNA repression.

Over 5400 miRNAs have now been identified with each miRNA possessing the ability to target multiple gene transcripts. miRNAs are members of complex gene regulatory networks (GRNs) and figure 3 summarises these GRNs, comprising of feedback and feed-forward loops.^66^ ^67^ ^69^ Certain subcircuits are evolutionarily favoured and are termed network motifs.^67^ Coordinated transcriptional and miRNA-mediated gene regulation is a recurrent network motif and fortifies gene regulation in mammalian genomes.^66^ Inflammation driven miRNA circuits are described in the literature and examples include nuclear factor-κB (NFκB) and hepatocyte nuclear factor-4α circuits.^70^ ^71^ Within the NFκB circuitry, transient activation of Src oncoprotein triggers an NFκB mediated inflammatory response by downregulating let-7a and upregulating its direct target interleukin (IL)-6.^70^ This forms a stable positive feedback circuit across many cell divisions.^70^ Similarly the hepatocyte nuclear factor-4α circuit consists of miR-124, IL6R, STAT3, miR-24 and miR-629 and is essential for liver development and hepatocyte function.^71^ Several other examples of miRNAs involved in GRNs are summarised in a recent review.^72^

### Regulation of miRNAs through epigenetic mechanisms

Emerging evidence suggests miRNA expression can be regulated by epigenetic mechanisms such as DNA methylation, histone modifications and circular RNAs (circRNAs).^73–76^ DNA methylation, the addition of methyl groups at CpG islands by DNA methyltransferases (DNMTs), is associated with transcriptional repression. Similarly, acetylation or deacetylation of histones may alter transcriptional activity.^77^ The recently established EpimiR database has collected 1974 regulations between 19 types of epigenetic modifications and 617 miRNAs across seven species.^78^ Aberrant DNA methylation of miRNAs has been demonstrated in various cancers, including lymphoid, gastric and colorectal malignancies.^79–81^ Up to 10% of miRNAs are tightly controlled by DNA methylation as seen in cell lines deficient in DNMT1 and DNMT3b.^82^ Time-dependent miRNA regulation has also been described. In murine models, partial hepatectomy results in downregulation of miR-127 as early as 3 h post partial hepatectomy with significant downregulation seen at 24 h.^83^ DNA methylation has also been shown to alter chromatin signatures and influence miRNA expression in cancer.^73^ Within the context of IBD, our group has studied epigenome-wide whole-blood DNA methylation profiles in treatment-naïve children with CD and healthy controls using the Ilumina 450 K platform.^7^ Sixty-five differentially methylated CpG sites achieving epigenome-wide significance were identified. The most significantly differentially methylated region in patients with CD involves the transcription start site for miR-21. Hypomethylation of the miR-21 locus in cases correlated with increased primary miR-21 expression in leucocytes and in inflamed intestinal mucosa.^7^

There appears to be a complex interplay between DNA binding proteins, chromatin modifications and miRNA expression. miR-155 assists in the differentiation and cytokine expression of Th17 cells as miR-155 deficient Th17 cells exhibit increased expression of Jarid2 which actively recruits polycomb repressive complex 2 to chromatin. Binding of polycomb repressive complex 2 to chromatin along with H3K27 histone methylation results in downregulation of cytokines IL-9, IL-10, IL-22 and Atf3.^84^

Recently circRNAs have been identified as regulators of miRNA expression. These endogenous RNAs can operate as miRNA sponges and are abundant within the human transcriptome.^85^ Hansen *et al*^76^ identified circRNA sponge for miR-7 as a potent inhibitor of miR-7 activity that is abundant in the mouse brain. circRNA sponge for miR-7 contains 70 highly selective miRNA target sites, strongly associated with AGO proteins and is highly resistant to miR-7 mediated destabilisation. They also identified testis specific sex determining region Y (*Sry)* circRNA as a miR-138 sponge indicating that the sponge effects of circRNAs are a general phenomenon.

**miRNA regulation**

- miRNAs are an integral part of GRN and modulate gene expression in response to cellular or environmental changes.
- Epigenetic mechanisms such as DNA methylation, histone modifications and circRNAs regulate the expression of miRNAs adding a layer of complexity to the regulation of gene expression.

## miRNA and the immune system

miRNAs are integral in differentiation, regulation and cell signalling, in the innate and adaptive immune system.^86^ ^87^ Maladaptation within these processes may result in acute or perpetuating inflammation, which characterises inflammatory disorders including IBD. Here key findings of the role of miRNAs in the innate and adaptive immune system are summarised, focusing on the most extensively investigated pathways.

### miRNAs and activation of the innate immune system

The innate immune system is the first defence against pathogens and relies primarily on early antigen recognition and this is initiated by pathogen associated molecular patterns. Pathogen associated molecular patterns trigger extracellular receptors termed toll-like receptors (TLRs) or intracytoplasmic nucleotide-binding oligomerisation domain-containing protein (NOD)-like receptors and promote downstream signalling cascades through pathways including NFκB, mitogen activated protein kinase and interferon (IFN) regulatory factors.^88^ miRNAs actively regulate these processes.

#### NOD-like receptors

Most relevant within the context of IBD is *NOD2,* part of the NOD-like receptors family. *NOD2* has been the strongest single genetic susceptibility locus in CD.^89^ The miRNA-NOD2 interaction has been studied and miRNAs including miR-192, miR-122, miR-29 and miR-146a may be implicated in IBD.^9^ ^90–92^ The interaction of miR-192 and NOD2 may be relevant in the pathogenesis of IBD as a SNP rs3135500 in the 3′UTR region of NOD2 reduces the ability of miR-192 to inhibit NOD2.^92^ Polymorphisms in *NOD2* can also impair the ability of dendritic cells (DCs) to express miR-29, resulting in exaggerated IL-23 induced inflammation.^9^ miR-122 has also been shown to target NOD2 expression upon LPS stimulation, albeit in a different cell line (HT-29 cells).^90^ Finally, miR-146a may regulate NOD2 derived gut inflammation in IBD and promote proinflammatory cytokines in MDP activated macrophages.^91^

#### Toll-like receptors

miRNAs have been shown to target a vast array of molecules within the TLR signalling pathway.^93^ miR-146a/b and miR-155 are the most relevant miRNAs in this field and their important regulatory activity is supported by their respective knockout (KO) mice phenotypes.^94^ ^95^ Mice deficient of miR-146a develop autoimmune disorders, myeloid cell proliferation and tumorigenesis while mice deficient of miR-155 display an impaired DC function and are unable to mount an adaptive immune response to pathogens.^94^ ^95^ The induction of the miR-146 family and miR-155 is nuclear factor κ light chain enhancer of activated B cells (κκB) dependent and these miRNAs form negative feedback circuits to fine-tune the inflammatory response upon bacterial infection.^96–99^

While miR-146 targets MyD88 adaptor proteins: tumour necrosis factor receptor associated factor 6 and IL-1 receptor-associated kinase 1, miR-155 on the other hand targets signalling proteins: suppressor of cytokine signalling 1 and TAK1-binding protein 2.^96^ ^100^ ^101^ Cells use miR146 to attain tolerance to subinflammatory doses of LPS, however when exposed to proinflammatory doses of LPS, miR-155 is also activated to broadly limit inflammation.^102^ The process of miR-146a expression appears dynamic and during early phases of the inflammatory response in macrophages, there is transient reversal of miRNA mediated repression of inflammatory cytokines through AGO2 phosphorylation.^103^ LPS stimulation of TLR4 also activates the regulatory PI-3K/Akt circuit which consists of let-7e and miR-155 and its targets TLR4 and suppressor of cytokine signalling 1.^104^ Macrophages deficient of Akt suppress let-7e and overexpress miR-155 resulting in a hyper-responsive phenotype to LPS.^104^ ^105^ miRNAs have also been implicated in other infections such as *Pseudomonas aeruginosa* infection promoting miR-302b expression in order to limit the pulmonary inflammatory response and BCG triggered miR-12 expression.^106^ ^107^

It must be borne in mind that the implicated miRNAs in the innate immune response are cell-specific. In human monocytes and neutrophils, TLR4 activated NFκB induces the expression of miR-9 however in murine macrophages, the NFκB feedback circuit is governed by miR-210.^108^ ^109^

### Adaptive immune system

**Figure 4:**
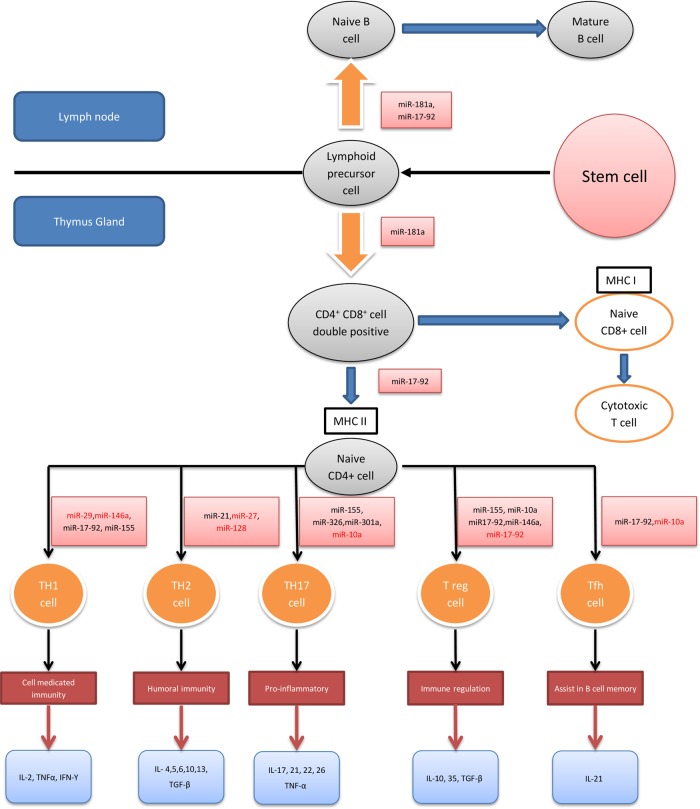
MicroRNAs (miRNAs) and the adaptive immune system. This diagram displays a developmental flow chart of the adaptive immune system, mainly T cells. The miRNAs highlighted in black promote the differentiation and/or function of their respective T cell populations while those highlighted in red are inhibitors of these processes. Cytokines released by each T cell subtype are also summarised.^94^ ^99^ ^100^ ^110–131^ MHC, major histocompatibility complex; Th, T helper cell; Tfh, T follicular helper cell; Treg, regulatory T cell; IL, interleukin; TNF, tumour necrosis factor; IFN, interferon; TGF, transforming growth factor.

Within the immune system, an intricate network of signalling facilitates maturation of the adaptive immune system. The appropriate development and function of these immune cells (T and B cells) is crucial when distinguishing foreign antigens from self. Recent studies have shown that miRNAs are involved in various stages of T cell and B cell maturation and activation (figure 4).

#### miRNAs and T cell regulation

The differentiation and maturation of T cells is influenced by miRNAs (figure 4). Specific deletion of Dicer or Drosha in T cell lineages results in aberrant differentiation and cytokine production with a marked bias towards Th1 development and IFN-γ production.^132^ ^133^ During positive and negative selections within the thymus gland, self-reactive T cells are first removed (negative selection) before T cells with functional receptors are selected (positive selection). The miR-181 family plays an important role in this process by altering T cell receptor sensitivity and may also contribute to diminished vaccine responses seen in the elderly.^110^ ^111^

#### miRNAs and Th1 and Th2 differentiation

miRNAs contribute to Th1 and Th2 cell differentiation. Several miRNAs including miR-146a, miR-29, miR-155, miR-17-92 cluster, miR-128 and miR-27b have been shown to influence Th1 differentiation and function.^112–115^ Overexpression of miR-155 influences CD4+ T cells to differentiate into Th1 cells while deficiency in miR-155 shows a bias towards Th2 differentiation.^94^ ^99^ Similarly, miR-17-92 promotes Th1 differentiation by upregulating IFN-γ production and suppressing regulatory T cell (Treg) differentiation.^116^ Of particular interest is the role of miR-21 expression in T cells. miR-21 has been shown to promote Th2 cell differentiation and as described previously, its dysregulation has been implicated in IBD.^5^ ^8^ ^117^

Several miRNAs have been shown to play a regulatory role by targeting transcription factors known to be involved in Th1 cell gene expression.^114^ These include miR-29 targeting T-bet and eomesodermin, transcription factors known to regulate IFN-γ production and miR-146a that targets signal transducer and activator transcription 1 in Treg cells, a transcription factor that controls Treg mediated regulation of Th1 responses.^113^ ^114^

#### miRNAs and Th17 differentiation

The Th17 pathway has been widely researched in the context of IBD.^134^ Recent studies determining the effect of miRNAs on the differentiation and function of Th17 pathway have identified direct and indirect regulatory mechanisms. Using murine models with experimental autoimmune encephalomyelitis, studies have shown that miR-326, miR-10a, miR-155 directly regulate Th17 differentiation and/or function while miR-301a is an indirect enhancer of Th17 differentiation.^118–121^ Of these miRNAs, miR-155 seems relevant to IBD as it directly upregulates Th17 differentiation and indirectly influences the regulation of pro-Th17 cytokines from DCs.^121^ ^122^ Furthermore, miR-155 KO mice are protected from dextran sulfate sodium(DSS) induced experimental colitis compared with wild type mice.^123^ miRNAs may also regulate hypoxia-induced Th17 differentiation by overexpressing miR-210 and promoting a negative feedback circuit with Hif1a, a key transcription factor of Th17 polarisation.^124^

#### miRNAs and T regulatory cells

Studies have identified the role of miRNAs in Treg cell development and function by promoting the differentiation of CD4^+^ T cells into Treg cells in the thymus and maintaining their immune homoeostatic function.^135^ It has been shown in vivo that CD4^+^ T cells that fail to express miRNAs develop spontaneous autoimmunity.^135^ Furthermore, conditional Dicer or Drosha deletion in Foxp3^+^Treg cells can alter the expression of several Treg specific markers including Foxp3, resulting in early fatal autoimmune disease.^133^ ^136^ Several miRNAs including miR-155, miR146a, miR-10a and miR-17-92 have been shown to maintain Treg cell function by modulating different signalling pathways.^100^ ^113^ ^125^ ^126^ miR10a in selective Treg cells assists in maintaining high Foxp3 levels but does not influence the number or phenotype of Treg cells.^125^ miR-155 has been shown to regulate mature Treg cell homoeostasis via the IL-2 signalling pathway while miR-146a regulates Treg cell function to limit inflammation.^100^ ^113^ The miR17-92 cluster has also been implicated in Treg cell function but these studies are conflicting. miR-17-92 Treg cell KO mice develop an exacerbated experimental autoimmune encephalomyelitis,^126^ however Jiang *et al* showed that certain miRNAs within this cluster such as miR17 and miR-19b inhibit Treg cell differentiation and promote Th1 responses.^116^

#### miRNAs and other immune cells

miRNAs have been implicated in other immune cell maturation such as B cells and T follicular helper cells. The miR-17-92 cluster helps regulate T follicular helper cell differentiation as well as B cell maturation while other miRNAs such as miR-10a and miR-181a have also been shown to regulate these processes.^119^ ^127–131^

**miRNAs and the immune system**

- miRNAs play important roles in the development and differentiation of the innate and adaptive immune system.
- The innate immune response to bacterial infection is regulated by an intricate network of miRNA circuits that fine-tune the inflammatory response.
- miRNA expression is highly cell specific and miRNA dysregulation especially in Th17 cells has been implicated in IBD.

## miRNA profiling in IBD

Following numerous studies determining miRNA expression profiles in human health and disease, researchers are now beginning to explore the functional actions of miRNAs. The various experimental techniques are used to investigate miRNAs and have been reviewed recently.^137^ Early studies used quantitative PCR (qPCR), following which microarrays have been used to study miRNAs. Microarrays work by hybridisation of the mature miRNA to complementary probe sequences immobilised on a chip or beads, with a detection mechanism usually involving labelling of the 3′ end of the miRNA.^137^ Most recently next generation sequencing (NGS) has been used to study small RNAs. NGS is potentially advantageous over microarray techniques as it provides greater coverage, demonstrates sequence independence and has the potential to identify novel miRNAs. Confirmatory techniques to validate these findings use standard techniques such as qPCR and northern blotting.

Results from early studies exploring the profile of miRNAs expressed in tissues of patients with IBD have been somewhat conflicting and difficult to interpret. Many of the miRNA related IBD studies have been underpowered and the need for large cohorts to perform well-powered studies has been demonstrated by the Cancer Genome Atlas consortia.^138^ There has also been a lack of uniformity of the comparator group, with controls consisting of healthy individuals in some studies and ‘symptomatic control’ patients in others and furthermore many of these studies used different methods to normalise miRNA data. There has been difficulty identifying suitable housekeeping genes as a reference for qPCR. Microarray and NGS studies have used different techniques for normalisation; either normalising against total miRNA or using approaches such as scaling and quantile normalisation.^139–141^

Other specific issues include difficulties deciphering whether differentially expressed miRNAs are causal, a consequence of disease or related non-specifically to inflammation, and miRNA levels may vary with disease duration and can be influenced by therapy.^142^ ^143^ Moreover, every cell type possesses its own unique epigenetic signature therefore interpreting the relevance of miRNAs detected in heterogeneous samples (eg, whole blood, intestinal biopsies) is challenging and complicated by the fact that many miRNAs can target the same gene. Recent publications have demonstrated a shift in focus from generating exhaustive tissue and blood miRNA screens (see online supplementary table S1) to carefully designed functional experiments that elaborate actions of individual miRNAs in known pathogenetic pathways in IBD as implicated by GWAS. The most consistent evidence to date links miRNAs and autophagy in CD and in NOD2-induced Th17-mediated disease (table 1).

**Table 1:** Functional studies on micro RNAs (miRNAs)

First author	Study model	miRNA of interest	mRNA/pathway target	Findings
Nguyen^144^	AIEC infection in T84 cells and mouse enterocytes. Translational studies in ileal CD biopsies	miR-30C and miR-130A	ATG5 ATG16L1	Adherent * Escherichia coli* upregulate miRNAs, reduce levels of ATG5 and ATG16L1 and inhibit autophagy
Zhai^145^	Jurkat T cells Colonic epithelial cells	miR-142-3p	ATG16L1	Reduced ATG16L1 mRNA and protein levels, regulating autophagy in CD.
Lu^146^	HCT116, SW480, HeLa and U2OS cell lines. Colonic biopsies from CD and healthy controls	miR106B, miR93	ATG16L1	Reduced levels of ATG16L1 and autophagy
Brest^10^	HEK-293 cells Colonic biopsies	miR-196	IRGM	A risk variant of *IRGM* alters the binding site for miR-196 and causes deregulation of *IRGM*-dependent xenophagy in CD
Brain^9^	Dendritic cell line, miR-29 KO murine models	miR-29	IL-12p40 (direct target) IL-12p19 (indirect target)	*NOD2* induces miR-29 release and limits IL- 23 release *NOD2* polymorphism alters the expression of miR-29 and contributes to pathogenesis in CD.
Xue^147^	IL-10 KO mice MyD88 KO mice RAG KO mice Murine dendrite cells	miR-10a	IL-12/IL-23p40	miR-10a expression is regulated by the intestinal microbiota and targets the Th17 pathway. This miRNA may play a role in intestinal homoeostasis
Koukos^148^	HCT-116 colonocyte cells, murine models and colonic biopsies from patients with UC	miR-124	STAT3	Downregulation of miR-124 results in proinflammatory response in UC
Shi^5^ Yang^149^	miR-21 KO DSS model and wild type mouse models Caco-2 cells, colonic biopsies from UC and healthy controls	mir-21	RhoB	miR-21 is overexpressed in inflammation and tissue injury. miR-21 KO improves survival in DSS colitis mouse model Targeting RhoB impairs the tight junction integrity and decreases transepithelial resistance and increases inulin permeability
Nata^150^	IL-10 deficient mice	miR-146b	siah2	miR-146b improves intestinal inflammation and epithelial barrier by activating NFκB
Chuang^92^	HCT116 colonic epithelial cells	miR-192, miR-495, miR-512, miR-671	NOD2	Downregulates NOD2 expression, suppresses NFκB, inhibits *IL8* and *CXCL3* expression
Chen^151^	Intestinal epithelial cells Intestinal biopsies	miR-200b	TGFβ	miR-200b promotes the growth of intestinal epithelial cells by inhibiting epithelial-mesenchymal transition via TGFβ
Feng^152^	UC intestinal biopsies HCT116 cells HT29 cells	miR-126	IκBα	Promotes NFκB mediated inflammation by targeting IκBα, a known inhibitor of the NFκB pathway.
Nguyen^153^	Caco2-BBE cells Mouse epithelial cells Colonic CD tissues	miR-7	CD98 expression	miR-7 regulates the expression of CD98. CD98 expression upregulated and miR-7 decreased in actively inflamed CD tissues.
Singh^123^	miR-155 KO mice	miR-155	Th17 pathway	miR-155 KO mice models and demonstrated that these mice are protected from experimental colitis compared with wild type mice.
Wu^8^	miR-21 KO TNBS and T cell transfer model of colitis miR-21 KO DSS colitis model	miR-21	Th1 pathway	miR-21 KO results in reduced DSS induced colitis but exacerbated inflammation in TNBS and T cell transfer model of colitis

AIEC, adherent invasive *Escherichia coli*; CD, Crohn's disease; DSS, dextran sulfate sodium; IL, interleukin; IRGM, immunity related GTPase family M protein; KO, knockout; miR, microRNA; NFκB, nuclear factor-κB; NOD2, nuclear oligomerisation domain-containing protein 2; TGFβ, transforming growth factor β; TNBS, trinitrobenzene sulfonic acid.

**miRNA profiling in IBD**

- There is a need to perform large-scale multicentre miRNA profiles in IBD with a well-defined ‘healthy control’ population using NGS techniques.
- miRNA levels can vary with disease duration and therapies.
- Every cell possesses its own epigenetic signature, therefore understanding the relevance of miRNA profiles in whole blood and intestinal biopsies can be challenging.

## Functional studies in IBD

### miRNAs and autophagy in CD

**Figure 5:**
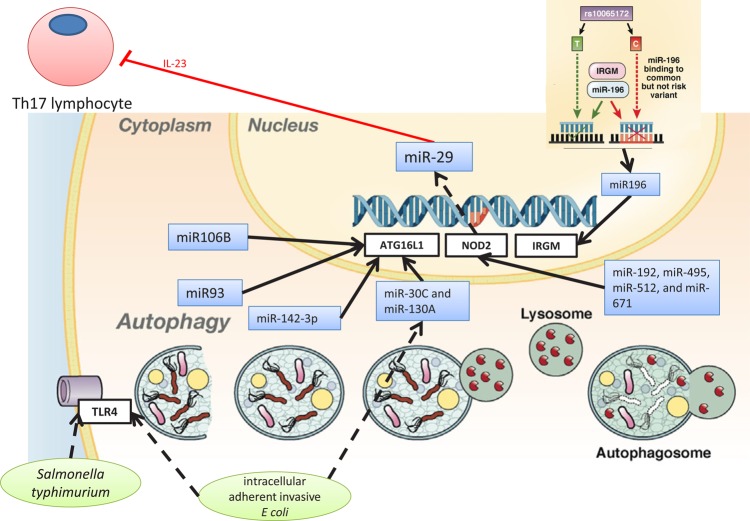
MicroRNAs (miRNAs) and autophagy (adapted with permission from Ventham *et al*, *Gastroenterology*). This diagram summarises the influence of miRNAs within different components of autophagy. Altered sequence in the immunity related GTPase family M protein (IRGM) gene results in an impaired binding site for miR-196.^10^ The consequent reduction in miR-196 activity results in IRGM upregulation and causes ineffective bacterial clearance of adherent invasive *Escherichia coli* (AIEC) in the intestinal cells of patients with Crohn's disease. ATG16L1 has also been shown to be a target of a host of miRNAs. miR-106B and miR-93 repress *ATG16L1* mRNA translation, thereby disrupting the autophagy pathway and bacterial clearance of AIEC.^146^ miR-30C and miR-130A have also been shown to directly target ATG16L1 and ATG5.^144^ Similarly, miR-142-3p has also been shown to negatively regulate *ATG16L1* and autophagy.^145^ Finally, NOD2 has been shown to induce the expression of miR-29 to limit IL-23 release, indirectly influencing the Th17 pathway in human dendritic cell lines.^9^ Polymorphisms in *NOD2* impair the ability to express miR-29 resulting in exaggerated IL-23 induced inflammation. Recently, a set of miRNAs that directly target NOD2 expression, miR-192, miR-495, miR-512 and miR-671 have also been described albeit in a different cell line (colonic epithelial HCT116 cells).^92^

Autophagy is a cellular process that involves self-digestion of unwanted materials such as damaged mitochondrial products (mitophagy) and pathogenic microbes (xenophagy). A process such as xenophagy requires the coordinated action of a multitude of proteins including, vimentin, NOD2, immunity related GTPase family M protein (IRGM) and a multiprotein complex which includes ATG16L1 and ATG5–ATG12.^154^ ^155^ In understanding molecular signalling and its effect on autophagy, several groups have investigated the role of miRNAs in these processes (figure 5).

During periods of starvation or hypoxia, mammalian target of rapamycin is inhibited within cells, activating autophagy. Hypoxia-induced autophagy results in upregulation of miR-155 that targets multiple components of mammalian target of rapamycin signalling.^156^ All genes currently described in the regulation of different stages of autophagy are influenced by miRNAs.^157^ Several autophagy genes have also been associated with susceptibility to CD, notably *IRGM* and *ATG16L1*.^158^ Interestingly, autophagy regulates miRNA production by targeting miRNA-processing enzymes Dicer and AGO2 through the autophagy receptor nuclear dot protein 52 kDa and gem-associated protein 4.^159^ Future challenges include understanding the genetic control of miRNA biogenesis including its own transcriptional activators and repressors.

### Immunity related GTPase family M protein

A common exonic synonymous SNP (c.313C>T) in *IRGM* is associated with CD.^160^ Although this SNP does not alter the IRGM protein sequence, it is located in the ‘seed’ region where mRNA and miRNA form a RNA induced silencing complex.^10^ Further analysis revealed that this SNP altered the binding site for miR-196. miR-196 was also shown to be overexpressed in inflamed tissues of patients with CD suggesting that this defective miRNA-mRNA interaction deregulates IRGM-dependent xenophagy in CD.^10^

### ATG16L1

GWAS identified *ATG16L1* polymorphism (T300A) as a risk variant in CD. Further studies revealed that this variant results in ineffective xenophagy of pathogens such as *Salmonella typhimurium*.^161^ Several studies have identified miRNAs that target ATG16L1, although each study associates a different set of miRNAs which may relate to miRNA cell line specificity. In HeLa cell lines, adherent invasive *Escherichia coli* infection results in overexpression of miR-93 and miR106B and downregulation of ATG5 and ATG16L1 thereby disrupting the autophagy pathway and bacterial clearance.^146^ In adherent invasive *Escherichia coli* infected T84 cells however, miR-30C and miR-130A were upregulated.^144^ Both studies were able to replicate their findings in endoscopic biopsies from patients with CD. Finally, miR-142-3p has also been shown to target ATG16L1 and autophagy using a different cell line.^145^

### Th17 pathway

Th17 driven inflammation plays an important role in IBD and studies have shown how miRNAs are used by DCs to regulate the inflammatory response. Brain *et al*^9^ demonstrated that NOD2 mediates its effects through miRNAs in DCs, in particular miR-29. The gene most strongly regulated by miR-29 is *IL12B* (encoding IL-12/23 p40) while *IL23A* (encoding IL-23 p19) is indirectly targeted through suppression of its transcription factor ATF2 and mice deficient of this miRNA develop a more severe Th17 driven colitis on DSS exposure.^9^ Microbiota can also impact on DC miRNA expression. In vivo models have demonstrated the commensal bacteria can negatively regulate miR-10a in DCs.^147^ Furthermore miR-10 directly targets IL-12/23p40 to limit Th17 driven inflammation and the expression of this miRNA may be regulated in order to maintain intestinal homoeostasis.^147^

### Other inflammatory pathways

The role of the NFκB pathway in IBD has been well described and studies have shown that this pathway is also regulated by miRNAs.^162^ miR-126 promotes NFκB mediated inflammation by directly targeting IκBα mRNA, an important inhibitor of NFκB signalling pathway. These findings were replicated in colonic biopsies in patients with active UC.^152^ Conversely, NFκB has also been shown to play an anti-inflammatory role in IBD as demonstrated by the differential expression of miR-146b in IL-10 deficient mice models.^150^ Administering miR-146b vectors intraperitoneally in DSS colitis mice ameliorated intestinal inflammation via activation of the NFκB mediated pathway.^150^

Other pathways that have been studied include signal transducer and activator transcription 3 (STAT3) and acetylcholine.^148^ ^163^ ^164^ Koukos *et al*^148^ demonstrated downregulation of miR-124 and upregulation of STAT3 in colonic biopsies of patients with active UC. These findings were translated in cell lines and murine experimental models suggesting a role of STAT3 expression in promoting intestinal inflammation. Finally, vagal secretion of acetylcholine suppresses peripheral inflammation by interrupting cytokine production and miR-132 has been shown to target acetylcholine esterase thereby potentiating anti-inflammatory effects.^164^

### Epithelial barrier integrity

Dysfunctional epithelial barrier has been implicated in the pathogenesis of UC.^165^ ^166^ GWAS data demonstrated IBD associated genes that play a role in maintaining intestinal epithelial barrier integrity and examples include *LAMB-1* that regulates basement membrane stability and *CDH-1* that regulates stability of adherens junctions via E-cadherin.^166^ Recent studies have investigated miRNAs in epithelial barrier function, in particular miR-21 and miR-200B.^5^ ^149^ ^151^ Murine miR-21 KO models with experimental DSS colitis survive longer and have less tissue inflammation than wild type mice and this miRNA targets *Rhob,* a target gene involved in regulating intestinal permeability.^5^ ^149^ Similarly miR-200b has been shown to help maintain epithelial barrier integrity by targeting transforming growth factor β1 and inhibiting epithelial-mesenchymal transition, a process that promotes loss of intestinal epithelial cells and contributes to the pathogenesis in IBD.^151^

**miRNA studies in IBD**

- miRNAs have been shown to regulate several pathways involving susceptibility loci found in IBD by GWAS.
- Recent data implicate miRNAs in the dysregulation of autophagy and Th17 signalling in CD.
- Increased expression of miR-21 is the most consistently replicated finding and represents a novel therapeutic target.
- miRNAs have also been shown to regulate intestinal barrier integrity in UC.

## Translation to clinical practice: lessons learned from other diseases

### miRNAs as disease biomarkers

Insights from contemporary cancer research highlight the exciting potential of miRNAs as biomarkers. Research in this area was stimulated by the initial finding that miRNA profiles can accurately differentiate between different cancer lineages and successfully classify poorly differentiated cancers based on tissue profiling.^167^ In 2008, miRNAs were also discovered to be present in serum in a cell-free state, sparking excitement about their potential use as non-invasive biomarkers.^168–170^ Extracellular miRNAs have now been found in most biological fluids including serum, urine, tears, saliva and breast milk.^171^ ^172^ Packaged in vesicles consisting of microparticles, lipoproteins or RNA binding proteins, these miRNAs are very stable and protected from degradation.^168^ Their profiles have been studied in various diseases including cardiovascular diseases, cancer and neurological diseases.^173–175^

Despite the optimism that miRNAs may represent robust biomarkers, the results should be treated with some circumspection; recent reviews showed that up to 58% (n=47) of the reported tumour related miRNAs are not disease specific.^176^ Only 33% of the reported miRNAs in non-neoplastic diseases (n=139) were deemed biologically plausible and represented non-ubiquitous miRNA expression in disease-appropriate cell types.^177^

### The therapeutic application of miRNA modulation

miRNA related therapeutic intervention may involve either miRNA antagonists or miRNA mimics. Antagomirs, an example of miRNA antagonists, can be applied to allow gain of function within diseased states by introducing a chemically modified RNA that binds to the active miRNA of interest to inhibit its activity and rescue the repression of its target. Conversely miRNA mimics are used to restore a loss of function by the introduction of miRNAs into diseased cells to mimic a healthy cell state.^178^

Within the GI literature, there are several studies highlighting the potential therapeutic application of specific miRNAs including miR-155 and miR-210.^123^ ^124^ Recently, miR-141 has been shown to play a critical role in colonic leucocyte trafficking by targeting CXCL12β. Treatment with pre-miR141protects mice against the development of trinitrobenzene sulfonic acid and IL-10KO colitis. In contrast, anti-miR141 aggravates trinitrobenzene sulfonic acid-induced colitis through CXCL12β suppression.^179^

Several miRNA-based therapies are either in the preclinical phase or the clinical trial phase, with ‘miravirsen’ miR-122 targeting in HCV being the most developed therapy.^180^ miR-122 is highly liver specific and well conserved across human and other vertebrate species.^181^ ^182^ The interaction between HCV and miR-122 is intriguing. The survival and replication of HCV RNA within the liver is propagated by miR-122 through two binding sites at the 5′ UTR of the HCV genome.^183^ Inhibiting this miRNA results in viral suppression, identifying it as a potential therapeutic target in HCV infection.^183^ Its antiviral effects have been demonstrated across all HCV genotypes.^184^ Data from the first Phase 2a clinical trial using miravirsen for the treatment of HCV (genotype 1) in 36 treatment-naïve patients with chronic HCV demonstrated dose-dependent reductions in viral RNA levels with no evidence of viral resistance or adverse events during an 18-week follow-up period.^180^ Combination therapies that include miravirsen with other known agents such as telaprevir and ribavirin are also currently in Phase 2 clinical trials.^185^ Several other miRNA therapies in development have been summarised in a recent review.^186^

### Challenges to therapeutic translation

There are several issues associated with miRNA therapeutics. First, there appears to be functional redundancy exhibited by miRNAs. Studies have shown that genetic deletion of miRNAs does not alter phenotypes or disease processes nor does it result in lethality in the vast majority of miRNAs. For some miRNAs that exist within ‘families’, this may be explained by intrafamilial redundancy however for others this may represent target sharing by several distinct miRNAs.^187^ ^188^ Temporary inhibition of miRNAs however seems to have an effect, as shown by the inhibition of miR-21.^189^ ^190^ The discrepancy in effect between permanent deletion and temporary inhibition of miRNAs may be a result of ‘off-target’ influences or could be explained by adaptive compensation by cells to chronic loss of functional miRNAs over time. Interestingly miRNAs may be particularly important under conditions of stress, such that miRNA deficient developmental phenotypes in controlled laboratory environments may not always be expected.^191^ ^192^

Uptake of miRNAs beyond the target organ poses a potential challenge when developing miRNA therapies aimed at overexpressing miRNA. For example miR-26a suppresses hepatocellular carcinoma but has also been shown to have pro-oncogenic properties in glioma formation by repressing its target, phosphatase and tensin homolog.^62^ ^193^ Second, miRNA-based drug delivery to the relevant cells must take into account the high rates of degradation by RNAses in blood.^194^ Finally, owing to a wide repertoire of several target genes, each miRNA-based therapy has the potential to cause varied side effects. Examples include germ line deletion of the oncogenic miR-17-92 cluster resulting in skeletal and growth defects in humans.^195^ As such the long-term inhibition of target miRNAs must be rigorously tested. Studies have shown that although short-term inhibition of miR-122 has beneficial effects on circulating cholesterol synthesis and repressing HCV replication in the liver, long-term inhibition of miR-122, as seen in KO mice models, results in an age-dependent increase in hepatocellular carcinoma and steatohepatitis.^196^ ^197^ These studies further emphasise the need to rigorously test short-term and long-term side effects of miRNA-based therapies.^195^

Delivery of miRNA therapies to their target organ has also been difficult. While antagomirs can be delivered systemically, the delivery of miRNA mimics has been challenging, similar to the difficulties encountered with small interfering RNA therapeutics. As single RNA strands are >10 times less effective in vitro and in vivo, miRNA mimics are delivered as synthetic duplexes.^198^ ^199^ There are however several issues that should be highlighted with this conformation. As mentioned earlier, cellular uptake can occur even in tissues that do not express the relevant miRNA, potentiating undesired effects. In addition, these RNA duplexes can also stimulate the innate immune system through TLRs.^200^ Finally, the passenger strands of these duplexes can incorporate themselves into miRNA induced silencing complexes and act as antagomirs with undesired side effects. Improvements in delivery strategies and RNA chemistries may combat some of these issues and miRNA replacement therapies for cancer using miRNA mimics have advanced to Phase 1 clinical trials.^201^ ^202^

There has also been much interest in studying innovative methods to deliver synthetic miRNAs. Studies have used lenti, adeno or adeno associated virus vectors to restore activity, however delivery using viral vectors certainly poses safety concerns.^62^ ^203–205^ The mechanisms of extracellular miRNAs packaged in vesicles are also being studied. Examples include exosomal delivery of small interfering RNAs to the mouse brain by systemic injection and exosomal delivery of let-7a to target epidermal growth factor receptor in RAG (−/−) mice.^206^ ^207^

**Translational application of miRNAs**

- There are emerging data from human diseases studying miRNAs as novel biomarkers in diagnosing and predicting disease course and response to therapy.
- miRNA-based therapeutic technologies have been restricted by difficulties in delivery to the target organ in order to minimise side effects.
- Several miRNA-based therapies are now in clinical or preclinical trials with ‘miravirsen’ being the most developed therapy.

## Concluding remarks: the immediate research agenda

The field of miRNA research has advanced dramatically with strong data associating miRNAs in IBD, notably in functional studies of autophagy and Th17 regulation. However in order to understand the role of miRNAs in disease pathogenesis, translational studies that take into account their plasticity and cellular specificity is critical. Novel miRNA biomarker discoveries are on the horizon, with studies using the dynamic properties of miRNAs to generate expression profiles in different stages of IBD and disease phenotype, or in response to immunomodulatory therapy.

Studies are now exploring miRNA regulatory and extracellular transport biology with a view to devising novel therapeutic targets that are cell specific and alter gene expression in target cells. The in vivo application of miRNA-based therapies packaged in genetically engineered extracellular vesicles provides a glimpse of the future translational potential of miRNA-based research in chronic inflammatory diseases.

## Supplementary Material

Web supplement
