# Supplementary material for: MicroRNAs: new players in IBD
Source: Gut. 2014 Dec 4;64(3):504–13. doi: 10.1136/gutjnl-2014-307891 (PMC4345829; doi:10.1136/gutjnl-2014-307891)
Supplement: Web supplement [file gutjnl-2014-307891-s1.pdf]

Supplementary table 1: Studies of microRNA expression

Authors	Cohort	Samples	miRNA expression	miRNA down-regulation
**Iborra**[w1]	9 active and 9 inactive CD<br>9 active and 9 inactive UC vs 33 healthy controls(serum only)	Serum and colonic biopsies	**Active vs inactive CD**<br>miR-188-5p, -877, -18a†, -629†, let-7b†, -140-3p†<br>**Active vs inactive UC**miR-188-5p, -877, -650†, 548a-3p†	**Active vs inactive CD**<br>miR-140-5p, -145, -18a, -128,-422a†, -885-5p†, -328†<br>**Active vs inactive UC**<br>miR-140-5p, -145, -18a, -128,-630†, -489†, -196b†
**Paraskevi** [w2]	128 CD and 88 UC vs 162 healthy controls	Peripheral blood	**CD**<br>miR-16, -23a, -29a, 106a, -107, -126, -191, -199a-5p, -200c, -362-3p, -532-3p<br>**UC**<br>miR-16, -21, -28-5p, -151-5p, -155, 199a-5p	
**Duttagupta** [w3]	20 active UC vs 20 healthy controls	Peripheral blood	**UC**<br>miR-188-5p, -378, -422a, -500,-501-5p, -769-5p, -874	
**Wu**[w4]	14 active CD vs 13 healthy controls<br>5 inactive CD vs 13 healthy controls<br><br>13 active UC vs 13 healthy controls<br>10 inactive UC vs 13 healthy controls	Peripheral blood	**Active CD**<br>miR-199a-5p, -340, -363-3p, -532-3p, miRplus-E1271<br>**Inactive CD**<br>miR-340<br>**Active UC**<br>miR-28-5p, -151-5p, -103-2, -199a-5p, -340, -362-3p, -532-3p, miRplus-E1271<br>**Inactive UC**<br>miR-28-5p, 103-2, 149, 151-5p, -340, -532-3p, and miRplus-E1153	**Active CD**<br>miR-149, miRplus-F1065<br>**Inactive CD**<br>miR149<br>**Active UC**<br>miR-505<br>**Inactive UC**<br>miR-505
**Zahm**[w5]	46 active CD vs 32 healthy controls	Serum	**active CD**<br>miR-16, -20a, -21, -30e, -93, -106a, -140, -192, -195, -484, let-7b	
**Bian**[w6]	5 active UC vs 4 healthy controls	Colonic biopsies	**active UC**<br>miR-150	
**Fasseu** [w7]	8 active UC vs 8 healthy controls<br><br>8 inactive UC vs 8 healthy	Colonic biopsies	**Active UC**<br>miR-7, -31, -135b, 223, 29a, 29b, -126, -127-3p, and -324-3p<br>**Inactive UC**<br>miR-196a, -29a, 29b, -126,	**Active UC**<br>miR-188-5p, -215, -320a, and -346<br>**Inactive UC**

	controls<br><br>8 active CD vs 8 healthy controls<br><br>8 inactive CD vs 8 healthy controls		-127-3b, and -324-3p<br>**Active CD**<br>miR-9, -21, -22, -26a, -29a, 29c, 30b, -31, -34c-5p, -106a, -126, -126, -127-3p, -130a, -133b, -146a, -146b-3p, -150 ,155, -181c, -196a, -324-3p, -375<br>**Inactive CD**<br>miR-9, -21, -22, -26a, 29b, 29c, 30a, -30b, -30c -31, -34c-5p, 106a, -126, -127-3p, -133b, -146a, 146b-3p, -150, -155, -196a -223, and -324-3p	miR-188-5p, -215, -320a, and 346
**Pekow**[w8]	8 active UC vs 8 healthy controls	Colonic biopsies		miR-143, -145
**Takagi**[w9]	12 active UC vs 12 healthy controls	Colonic biopsies	miR-21 , -155	
**Wu**[w10]	5 active CD vs 13 healthy controls<br>6 aSBCD vs 13 healthy controls	Colonic and SB biopsies	**Active CD(colonic)**<br>miR-23b, -106a, and -191<br>**aSBCD**<br>miR-16, -21, -223, and 594	**Active CD (colonic)**<br>miR-19b and -629
**Wu**[w11]	15 active UC vs 15 healthy controls	Colonic biopsies	miR-16, -21, 23a, 24, 29a, 126, 195, and left-7f	miR-192, 375, and 422b
**Lin** [w12]	19 with IBD vs 18 controls with diverticular disease	Colectomy tissues	miR-31, -206, -424, -146a	

miR† denotes miRNA expression in tissues. All studies used qRT-PCR and/or microarray techniques for miRNA expression except for Lin et al who performed next generation sequencing.

Abbreviations: CD: crohn’s disease, UC: ulcerative colitis, SBCD: small bowel crohn’s disease, miR: miRNA; IBD: inflammatory bowel disease

References

w1 Iborra M, Bernuzzi F, Correale C, *et al.* Identification of serum and tissue micro-RNA expression profiles in different stages of inflammatory bowel disease. *Clin Exp Immunol* 2013;**173**:250–8. doi:10.1111/cei.12104

w2 Paraskevi A, Theodoropoulos G, Papaconstantinou I, *et al.* Circulating MicroRNA in inflammatory bowel disease. *J Crohns Colitis* 2012;**6**:900–4. doi:10.1016/j.crohns.2012.02.006

w3 Duttagupta R, DiRienzo S, Jiang R, *et al.* Genome-wide maps of circulating miRNA biomarkers for ulcerative colitis. *PLoS One* 2012;**7**:e31241. doi:10.1371/journal.pone.0031241

w4 Wu F, Guo NJ, Tian H, *et al.* Peripheral blood microRNAs distinguish active ulcerative colitis and Crohn's disease. *Inflamm Bowel Dis* 2011;**17**:241–50. doi:10.1002/ibd.21450

w5 Zahm AM, Thayu M, Hand NJ, *et al.* Circulating microRNA is a biomarker of pediatric Crohn disease. *J Pediatr Gastroenterol Nutr* 2011;**53**:26–33. doi:10.1097/MPG.0b013e31822200cc

w6 Bian Z, Li L, Cui J, *et al.* Role of miR-150-targeting c-Myb in colonic epithelial disruption during dextran sulphate sodium-induced murine experimental colitis and human ulcerative colitis. *J Pathol* 2011;**225**:544–53. doi:10.1002/path.2907

w7 Fasseu M, Tréton X, Guichard C, *et al.* Identification of restricted subsets of mature microRNA abnormally expressed in inactive colonic mucosa of patients with inflammatory bowel disease. *PLoS One* 2010;**5**. doi:10.1371/journal.pone.0013160

w8 Pekow JR, Dougherty U, Mustafi R, *et al.* miR-143 and miR-145 are downregulated in ulcerative colitis: putative regulators of inflammation and protooncogenes. *Inflamm Bowel Dis* 2012;**18**:94–100. doi:10.1002/ibd.21742

w9 Takagi T, Naito Y, Mizushima K, *et al.* Increased expression of microRNA in the inflamed colonic mucosa of patients with active ulcerative colitis. *J Gastroenterol Hepatol* 2010;**25 Suppl 1**:S129–33. doi:10.1111/j.1440-1746.2009.06216.x

w10 Wu F, Zhang S, Dassopoulos T, *et al.* Identification of microRNAs associated with ileal and colonic Crohn's disease. *Inflamm Bowel Dis* 2010;**16**:1729–38. doi:10.1002/ibd.21267

w11 Wu F, Zikusoka M, Trindade A, *et al.* MicroRNAs are differentially expressed in ulcerative colitis and alter expression of macrophage inflammatory peptide-2 alpha. *Gastroenterology* 2008;**135**:1624–1635.e24. doi:10.1053/j.gastro.2008.07.068

w12 Lin J, Welker NC, Zhao Z, *et al.* Novel specific microRNA biomarkers in idiopathic inflammatory bowel disease unrelated to disease activity. *Mod Pathol* 2014;**27**:602–8. doi:10.1038/modpathol.2013.152
